# Prevalence and factors associated with intestinal parasites among under-five children attending Woreta Health Center, Northwest Ethiopia

**DOI:** 10.1186/s12879-019-3884-8

**Published:** 2019-03-13

**Authors:** Habtamu Sewunet Mekonnen, Daniale Tekelia Ekubagewargies

**Affiliations:** 1 Department of Medical Nursing, School of Nursing, College of Medicine and Health Sciences, University of Gondar, Gondar, Ethiopia; 2 Department of Pediatrics and Child Health Nursing, School of Nursing, College of Medicine and Health Sciences, University of Gondar, Gondar, Ethiopia

**Keywords:** Intestinal parasite, Prevalence, Under-five children, Ethiopia

## Abstract

**Background:**

Environmental, social, geographical, and other factors could affect the distribution of intestinal parasites. Parasitic infections would impose on health and social problems like mal-absorption, diarrhea, impaired work capacity, and reduced growth rate. However, there is a scarcity of information regarding the prevalence of intestinal parasites and associated factors in the study area.

**Methods:**

Institution based cross-sectional study was conducted among 310 study participants from April–May, 2017. Study participants were selected using a systematic random sampling technique. EPI Info version 7 and SPSS version 20 were used to enter and analyze the data. Both bivariate and multivariate logistic regression analyses were computed. In multivariate analysis, variables with *P*-value < 0.05 were considered statistically significant.

**Results:**

In this study, the mean age of participants was 29.25 Months. The overall prevalence of intestinal parasites was 18.7% (95% CI = 14.4–23.3). Children who rarely feed fresh meal (AOR = 7.74, 95% CI: 1.61, 7.84), Children whose nails were sometimes trimmed (AOR = 3.41, 95% CI: 2.20–10.28), children who had no clean playing ground (AOR = 2.43, 95% CI: 1.25–5.18), and children who had open defecation of the family (AOR = 3.40, 95% CI: 1.27–10.86) were significantly associated with intestinal parasitic infections. *Among the intestinal parasites,* 31(53.5%) were *G.lamblia (Giardia lamblia)* and 21(36.2%) were *E. histolytica/E. dispar/E. moshkovskii.*

**Conclusion:**

In this study, the prevalence of intestinal parasites was found low compared with the WHO annual or biannual population prevalence and treatment. However, strengthening of health education about food, personal and environmental hygiene of both children and mothers/guardians is crucial to limit the transmission. Besides, improving mothers/guardian awareness about the mode of intestinal parasites transmission is necessary.

## Background

Intestinal parasites primarily infect the gastrointestinal tract (GIT) but can live throughout the body. Helminths and protozoa are the two main types of intestinal parasites that live in the intestines [1].

Intestinal helminths and protozoan infections have been recognized as significant causes of illnesses and death worldwide [2]. These are among the most common human parasitic infections and have been associated with important morbidity and economic loss in endemic areas [3, 4]. Worldwide, more than one-sixth of the population is infected by intestinal parasites of which majority live in developing countries [5, 6]. Among intestinal parasitic infections, *Ascariasis*, *hookworm,* and *Trichiuriasis* are responsible for one billion, 900 million, and 500 million infections respectively, and cause significant morbidity and mortality [7].

The prevalence of intestinal parasites among under-five, preschool and school children were 17.7% in Riyadh, Saudi Arabia [8], 52.8% in an urban slum of Karachi, Pakistan [9], 19.6% in Zambia [10], and 30% in Khartoum, Sudan [11]. In Ethiopia its prevalence varies from area to area; in Wondo Genet 85.1% [12], Aynalem village, Tigray 48.1% [13], Debre Birhan referral Hospital 17.4% [14], Adare and millennium health center in Hawassa 26.6% [15], Wonji Shoa Sugar Estate 24.3% [16]. Intestinal parasitic infection accounts for a global health burden in developing countries mainly due to fecal contamination of water and food, climatic, environmental, and socio-cultural factors enhancing parasitic transmission [17–19]. In urbanized countries, protozoan parasites infection is in contrast to helminths. *Amoebiasis* is one of the most important reasons for death from parasitic diseases wide-reaching with its impact on people of developing countries [7].

The distribution of helminths and protozoan parasites are varied in different regions. In developing countries, enteric protozoa mainly *Giardia intestinalis including G.lamblia (Giardia lamblia)* and *Entamoeba* spp. were common in children. The systematic review and meta-analysis study on prevalence of gastrointestinal pathogens in developed and developing reviled *Giardia intestinalis* as the most frequently detected protozoa in developing regions, with the prevalence of 3.0 and 2.7% in South Asia and Sub-Saharan Africa respectively. The prevalence of *Entamoeba* spp. was 1.5% in Middle East, North Africa, and Sub-Saharan Africa. *Cryptosporidium* was 1.0 and 1.7% in the Middle East and North Africa, and in South Asia respectively. But *Dientamoeba fragilis,* was found in < 1% of cases in each region [20]. The prevalence of *G.lamblia (Giardia lamblia) and Entamoeba histolytica were (8.5, 5.7%) 17.46, 0.87%), and (3, 2%) in* Debre Birhan referral hospital, Southwestern Iran, and Taifg overnorate respectively [14, 21, 22].

Ethiopia has one of the lowest qualities of drinking water supply and latrine coverage in the world (https://en.wikipedia.org/wiki/Water_supply_and_sanitation_in_Ethiopia, [23–25]). The distribution and prevalence of various species of intestinal parasites differ from region to region because of several environmental, social and geographical and other factors. Mal-absorption, diarrhea, impaired work capacity, and reduced growth rate due to intestinal parasitic infections constitute important health and social problems. These infections are more prevalent among the poor segments of the population and intimately linked with low economic level, poor personal and environmental sanitation, and overcrowding, limited access to clean water, tropical climate and low altitude [26–29]. However, there is scarcity of information regarding the prevalence of intestinal parasites and associated factors in the study area. Therefore, this study aimed to assess the magnitude of intestinal parasite infection and its associated factors among under-five children attending in Woreta Health Center, Northwest Ethiopia.

## Methods

### Study design and period

An institution-based cross-sectional study was conducted from April–May, 2017.

### Study area

The study was conducted in Woreta health center at Woreta town. Woreta town is the capital town of Fogera district located in South Gondar zone, Amhara region, east of Lake Tana and south of Addis Zemen and 614 km northeast from Addis Ababa, the capital city of Ethiopia. Woreta is home of 42,595 inhabitants. Fogera has been known for its flat and low land. The altitude of the district ranged between 1750 and 2100 m above sea level. The mean annual rainfall of the woreda is 1216.3 mm and ranges from 1103 to 1336 mm. the climatic condition of Woreta town is tropical savanna climate with 20.3 c^0^ average temperature. The population in the town mainly lived with trade work, while the rural population lived with crop production and by raring chattels. There are two major rivers that are of great economic importance to the district and to the region which found in short distance to Woreta town. These rivers are mainly used for irrigation during the dry season for the production of horticultural crops, mainly vegetables. Some farmers also use water pumps to produce vegetables, cereals, and pulses.

Woreta health center provides health service for the town as well as a catchment area, the district. An average, 50 children were attending the under-five clinic per day.

### Source population

All under five children who attended Woreta health center.

### Study population

All under-five children who attended Woreta Health Center the during data collection period.

### Inclusion criteria

Under-five children attended in under- five clinic at Woreta health center during data collection.

### Exclusion criteria

Under-five children who started anti-parasitic drug/s and those who were critically ill.

### Sample size determination and sampling procedure

The sample size was calculated using a single population proportion formula ((n = [(Zα/2)2 × P (1-P)]/D2) (n = sample size, Zα = level of confidence, P = estimated proportion/prevalence of the population, D = tolerated margin of error) with the assumption of a 95% level of confidence, 5% margin of error, taking prevalence of 26.6% from the study conducted in Hawssa, Ethiopia [15] and with a 5% non response rate; the final sample size was 310. Study participants were selected using systematic random sampling and every third child was interviewed based on their order of arrival.

### Data collection tool and procedure

A questionnaire regarding the explanatory variables was prepared by intensively reviewing local and international literature. The questionnaire was first developed in English and translated to Amharic, the local language, and then retranslated back to English for analysis to ensure the consistency. A pre-test was administered on 16 under-five children at Addis Zemen health center, northwest Ethiopia. Some amendment was made on the tool after the pre-test. One BSc graduate nurse for supervision and collection of explanatory variables and two well-experienced laboratory technologists for stool specimen diagnosis were employed. The data collection technique was a face to face interview with their mothers/guardians by using a structured questionnaire using the Amharic language.

### Stool sample collection and examination

Single, 5 g of fresh stool specimens were collected from study participants in clean, labeled stool cups then direct wet mount technique was used. Kato-Katz thick smear method using delivering a plug of 50 mg of stool was processed in a portion of the sample and the residual was placed in vials having 10% formalin. Samples were qualitatively examined on the spot for hookworm ova and other intestinal helminthic infections.

Quantitative examination of the Kato-Katz slides for helminthiasis (except for hookworms) was done in the laboratory within one week of stool collection. But the Kato-Katz preparation was read immediately for hookworm parasite. Stool specimens placed in vials were also qualitatively examined in the laboratory for strongyloidiasis and protozoan parasites by the formol-ether concentration method. To ensure the quality of the investigation, the two readers read the slides independently and their readings were compared. Discordant were immediately resolved with a discussion of each other and in consultation with other experts.

### Data quality control technique

The questionnaires were prepared in English version then translate into the local language, Amharic for the interview and then to ensure the consistency, it was translated back to English. The tool was pretested one week prior to the actual data collection date. One day training was given for data collectors and supervisors about the questionnaire and data collection technique.

### Operational definition

**Intestinal parasites:** are parasites that can infect gastrointestinal tracts of the human body.

**Parasite infection:** intestinal parasite infection/positive result confirmed by laboratory stool examination.

**Utensils:** any materials/ handheld tools used to store/carrying, cook, and feed food.

**Clean playing ground:** surfaces with no visible dust, dirt, mud or contaminating particles on which children climb, slide, crawl, push, pull, swing and contact for playing purpose.

### Data processing and analysis

The data were checked for its completeness and coded manually before data entry. EPI Info version 7 and SPSS version 20 were used to enter and analyze the data. Descriptive statistics; including frequency, mean and standard deviations, were used to describe the data. Bivariate logistic regression analysis was carried out and variables having *P*-value of ≤0.2 were entered into multivariate logistic regression for final analysis.

## Results

### Socio-demographic characteristics of the participants

In this study, 15 children were excluded from the study by the exclusion criteria. A total of 310 under-five year children were included in the study with a response rate of 100%. The mean age of participants was 29.25 Months. More than half of the respondents 158(51%) were females. Majority of mothers/guardian religion 285 (91.9%) were Orthodox Christian and 250 (80.6%) were from Amhara ethnicity (Table 1).

**Table 1:** Socio-demographic characteristics of under-five children/mother/guardian in Woreta Health Center, Northwest Ethiopia, 2017 (*n* = 310)

Variables	Frequency	Percent (%)
Sex		
Male	152	49.0
Female	158	51.0
Age		
< 6 months	26	8.4
6 month- 11 month	50	16.1
12–23 months	63	20.3
24–59 months	171	55.2
Family Residence		
Urban	166	53.5
Rural	144	46.5
Religion of mother/guardian		
Orthodox	285	91.9
Muslim	25	8.1
Ethnicity of mother/guardian		
Amhara	250	80.6
Tigrie	60	19.4
Occupation of mother/guardian		
Government	71	22.9
Housewife	121	39
Merchant	72	23.2
Farmer	46	14.8
Mother/guardian educational back ground		
Unable to read and writing	79	25.48
Able to read and writing	69	22.3
Grade 1–8	68	21.9
Grade 9–12	32	10.3
Certified and above	62	20
Monthly family income		
< 1000 Et Birr	67	21.6
2000–3000 Et Birr	152	49
> 3000 Et Birr	91	29.4

### Characteristics of food, personal and environmental hygiene

In this study, the majority 282 (91.2%) of participants were had clean dinning utensils. More than half 166 (53.5%) and two-thirds 210 (67.7%) of mothers/caregivers wash their hand sometimes after toileting and trim their child’s nails when grown respectively. Only 178 (57.4%) of mothers/caregivers had tap water as a source of drinking water and 55 (17.7%) of mothers/caregivers knew both contaminated food and water as a mode of transmission of intestinal parasites (Table 2).

**Table 2:** Characteristics of food, personal and environmental hygiene of participants at Woreta Health Center, Northwest Ethiopia, 2017 (*n* = 310)

Variable	Frequency	Percent (%)
Are dinning utensils Clean		
Yes	282	91.0
No	28	9.0
Do you wash your hand after toilet before touching your child		
Always	144	46.5
Sometimes	166	53.5
Does your child eat unwashed fruits and vegetables		
Always	22	7.1
Sometimes	90	29.0
Never	154	63.9
Your child meal		
Always fresh	98	31.6
Sometimes fresh	183	59.0
Rarely fresh	29	9.4
Do you trim your child’s nails when grown		
Always	100	32.3
Sometimes	210	67.7
Does your child take other food before the age of six month		
No	286	92.3
Yes	24	7.7
Your child playing ground		
Not clean	110	35.5
Clean	200	64.5
What is your source of drinking water		
Tap water	178	57.4
Stream water	132	42.6
Type of your toilet		
Open defecation	138	44.5
Public	74	23.9
Private	98	31.6
Knowledge of mode of transmission		
Contaminated food	124	40
Contaminated water	131	42.3
Both	55	17.7

### Prevalence of intestinal parasitic infections

**Fig. 1:**
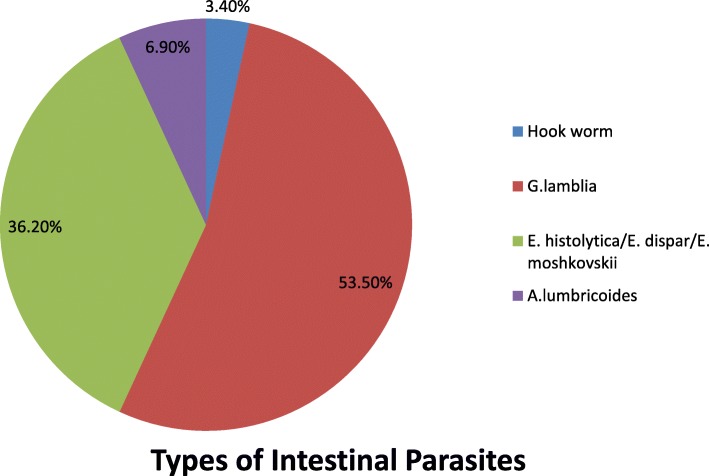
Types of intestinal parasites among under-five children attending at Woreta Health Center, Northwest Ethiopia, 2017 (*n* = 58)

The overall prevalence of intestinal parasitic infections among under-five children was 18.70% (95% confidence interval (CI) =14.4–23.3). Among the four identified intestinal parasites; the predominant were 31(53.5%) *G.lamblia (Giardia lamblia)* and 21(36.2%) *E. histolytica/E. dispar/E. moshkovskii* (Fig. 1).

### Factors associated with intestinal parasitic infections

Multivariate logistic regression analysis showed that child food freshness, regular trimming fingernails, children playground cleanliness, and the family use of toilet were significantly associated with intestinal parasitic infection.

Children who were rarely fed fresh meal were 7.74 (AOR = 7.74; 95% CI: 1.61, 7.84) more likely to be infected by intestinal parasites than children feed always a fresh meal. Children whose nails were trimmed sometimes were 3.41(AOR = 3.41; 95% CI: 2.20–10.28) more likely to be infected by intestinal parasites than children whose nails were cut always. Those children who have not clean playing ground were 2.43 times (AOR = 2.43, 95% CI: 1.25–5.18) more likely to be infected by intestinal parasites than children who had clean playing ground. Besides, children who had open defecation of the family were 3.4 times (AOR = 3.40, 95% CI: 1.27–10.86) less likely infected by intestinal parasites than children who had a family open defecation. However, there is no observed significant association between the dependent variable and age of children and other socio-demographic variables (Table 3).

**Table 3:** Bivariate and multivariate analysis for the prevalence of intestinal parasites under-five children attending Woreta Health Center, Northwest Ethiopia, 2017 (*n* = 310)

Variables	Parasite infection		COR (95% CI)	AOR (95% CI)
	Positive/Yes	Negative/No		
Child Age				
< 6 months	6	20	1	1
6–11 months	6	44	0.45 (0.65–4.5)	0.92 (0.40–4.96)
12–23 months	8	55	0.48 (0.27–1.44)	0.54 (0.51–1.42)
23–59 months	38	133	0.95 (0.68–3.57)	1.74 (0.41–4.22)
Family residence				
Urban	21	145	1	1
Rural	37	107	2.40 (1.12–6.10)*	2.64 (0.51–4.11)
Mother/Guardian Occupation				
Government	10	61	1	1
Housewife	34	87	2.33 (1.07–5.07)*	1.94 (0.37–10.15)
Merchant	8	64	0.76 (0.28–2.06)	0.55 (0.26–1.83)
Farmer	6	4	0.96 (0.32–2.87)	1.40 (0.36–5.34)
Mother/guardian educational status				
Unable to read and write	24	55	1	1
Able to read and write	13	56	2.77 (1.14–6.73)*	0.87 (0.34–2.59)
Grade 1–8	8	60	1.58 (0.60–4.08)	0.80 (0.15–4.26)
Grade 9–12	5	27	0.90 (0.32–2.56)	1.08 (0.22–7.27)
Certified and above	8	54	1.56 (0.49–4.96)	0.74 (0.03–17.52)
Family income				
< 1000 Et Birr	10	57	1.12 (0.29–3.99	1.98 (0.66–4.23)
2000–3000 Et Birr	37	115	2.08 (0.47–2.88)	3.48 (0.98–4.69)
> 3000 Et Birr	11	71	1	1
Are dinning utensils clean				
Yes	49	233	1	1
No	9	19	2.25 (0.96–5.27)	1.88 (0.66–5.38)
Do you wash your hand after toilet before touching your child				
Always	17	127	1	
Sometimes	41	125	2.46 (1.01–3.23)*	3.30 (0.92–4.27)
Does your child eat unwashed fruits and vegetables				
Always	8	14	4.71 (1.71–12.97)	2.31 (0.70–7.58)
Sometimes	34	56	2.65 (1.39–5.05)	1.72 (0.81–3.77)
Never	16	138	1	1
Your child meal				
Always fresh	5	93	1	1
Sometimes fresh	45	138	6.07 (0.56–4.07)	6.69 (0.16–3.56)
Rarely fresh	8	21	7.09 (1.35–8.13)*	7.74 (1.61–7.84)**
Do you trim your child’s nails when grown				
Always	7	93	1	1
Sometimes	51	159	4.26 (2.50–11.21)*	3.41 (2.20–10.28)**
Does your child take food other than breast milk before the age of six month				
Yes	51	235	0.53 (0.22–1.36)	1.28 (0.37–4.67)
No	7	17	1	1
Your child playing Ground				
Not clean	32	78	2.75 (1.53–4.95)*	2.43 (1.25–5.18)**
Clean	26	174	1	1
What is your source of drinking water				
Tap water	24	154	1	1
Stream	34	98	2.23 (1.24–3.26)*	2.55 (0.65–5.27)
Type of your toilet				
Open defecation	22	116	0.51 (0.22–0.67)*	3.40 (1.27–10.86)**
Public	5	69	0.16 (0.06–0.43)	3.83 (1.25–10.60)
Private	31	67	1	1
Knowledge about the mode of transmission				
Contaminated food	27	97	1	1
Contaminated water	23	108	1.64 (0.69–3.87)	1.41 (0.45–2.37)
Both	8	47	1.25 (0.52–2.99)	1.02 (0.35–2.95)

*Variables those were significant during bivariate logistic analysis at *P* value ≤ 0.05

**Variables that were found to have a significant association in multivariate analysis at *p*-value ≤ 0.05

## Discussion

It is of paramount importance to know the distribution and extent of intestinal parasitic infection in a given community to devise successful preventive and therapeutic interventions. This study assessed the prevalence as well as factors associated with intestinal parasite infection. The overall prevalence of intestinal parasitic infections among under-five children was 18.70% (95% confidence interval (CI) =14.4–23.3 with 31(53.5%) *G.lamblia* and 21(36.2%) *E. histolytica/E. dispar/E. moshkovskii* the most prevalent species.

The finding of this study was in-line with what was found in Wonji Shoa 24.3% and Addis Ababa 14.9% [16, 30]. This similarity could be due to the fact that both studies used similar study design and selection of participants.

But the current study showed a lower prevalence of intestinal parasite infection as compared to those studies conducted in Cuba 45.2% [31], Egypt 47.3% [32], Kenya 25.6% [12], Addis Ababa 27.5%, Wondogenet 85.1% [33]. This could be because of in the current study all children attending the health facility were included while in the study conducted in Kenya the investigators considered children with the diarrheal disease for the study which could increase the prevalence in the latter. In the case of a study in Cuba, investigators took three different samples from each participant and conducted three separate tests and this may increase the likelihood of the diagnosis of at least one intestinal parasitosis while in the case of Egyptian study it was conducted among squatter settlement which has no adequate infrastructure such as clean water source which also can increase the probability of acquiring parasitic infections.

In this study, it was shown that child food freshness, regular trimming fingernails, children playground cleanliness, and the use of toilet were significantly associated with intestinal parasitic infection.

Eating rarely fresh food was found to be a risk to intestinal parasites compared to those who were feed always fresh. This is because of the fact that storage of cooked food for longer period gives rise to a proliferation of bacteria and other parasites [34]. Irregular trimming of fingernails of children was also significantly associated with intestinal parasitosis. This result is supported by similar studies conducted in Ethiopia [35]. This could be attributed to the removal of accumulated dirt containing eggs of parasites on fingernails up on trimming [36]. For example, a study in Egypt found *E. vermicularis* and *H. nana* eggs in 2% of the fingernail clippings [37].

Children who had unclean playground were found to be at an increased rate of having intestinal parasites (AOR = 2.43, 95% CI: 1.25–5.18). This is due to the presence of microorganisms and eggs of parasites in dirty surfaces. This is supported by a study which concluded that inadequate sanitation and hygiene behavior are associated with soil-transmitted helminths and intestinal protozoa infections [38]. On the other hand, children who had open defecation of the family were 3.4 times more likely to harbor intestinal parasites (AOR = 3.40, 95% CI: 1.27–10.86). This finding is in line with a cluster randomized controlled trial conducted in India that showed the inversely proportional relationship between improved latrine use and intestinal parasitosis prevalence [39] and another study conducted in Côte d’Ivoire [38].

As to the limitation; Even though, molecular assays and other techniques could best estimate the prevalence of intestinal parasites and differentiating different specious of *ameabiasis*, in this study the microscopy technique was used to determine the prevalence.

## Conclusion

In this study, the prevalence of intestinal parasites was found lower than WHO annual or biannual population treatment level. Child food freshness, regular trimming fingernails, Children playground cleanliness, and family use of toilet were significantly associated with intestinal parasitic infection. Thus, strengthening of health education about food, personal and environmental hygiene of both children and mothers/guardians is crucial. Besides, improving mothers/guardian awareness about the mode of intestinal parasites transmission is necessary.

## Abbreviations

*A.lumbricoides*: *Ascaris Lumbericoides*
AOR: Adjusted odds ratio
CI: Confidence interval
COR: Crude odds ratio
*G.lamblia*: *Giardia lamblia*
GIT: Gastrointestinal tract
IP: Intestinal parasites
SPSS: a Statistical package for social sciences
WHO: World Health Organization
